# Supplementary material for: NBF2, an Algal Fiber-Rich Formula, Reverses Diabetic Dyslipidemia and Hyperglycemia In Vivo
Source: Int J Mol Sci. 2024 Oct 9;25(19):10828. doi: 10.3390/ijms251910828 (PMC11476984; doi:10.3390/ijms251910828)
Supplement: Supplementary file 1 [file ijms-25-10828-s001.zip › ijms-3189079-Supplementary.pdf]

Supplementary Table S1. Distribution of calorie intake by rat group (N=26)

Rat group	T1 [mean (SD)]	T2 [mean (SD)]	T3 [mean (SD)]	T4 [mean (SD)]	T5[mean (SD)]	T6 [mean (SD)]	T7 [mean (SD)]	p
OLETF cont	110 (0.7)	98.3 (1)	126.3(18)	101.1(8.7)	96.2(8.4)	117.2(1.7)	102.2(0.7)	-
HD-NBF2	99.5(1.2)	100.9(0.8)	128.2(25)	99.2(2.6)	98.5(1.2)	104.1(2.9)	96.4(4.7)	NS
LD-NBF2	105.2(5.8)	100.3(0.2)	125.5(8.3)	95.4 (2)	97.9(4.8)	114.7(6.2)	97.9(5.1)	NS
Normal cont	80.5 (5.2)	70.7 (0.7)	80.1 (5.6)	79.4 (3.1)	77.3(2.8)	78.4(2.4)	74.9(3.1)	**0.000**

Notes: NS, not significant; p, p-value; cont., control; T, measurement time.

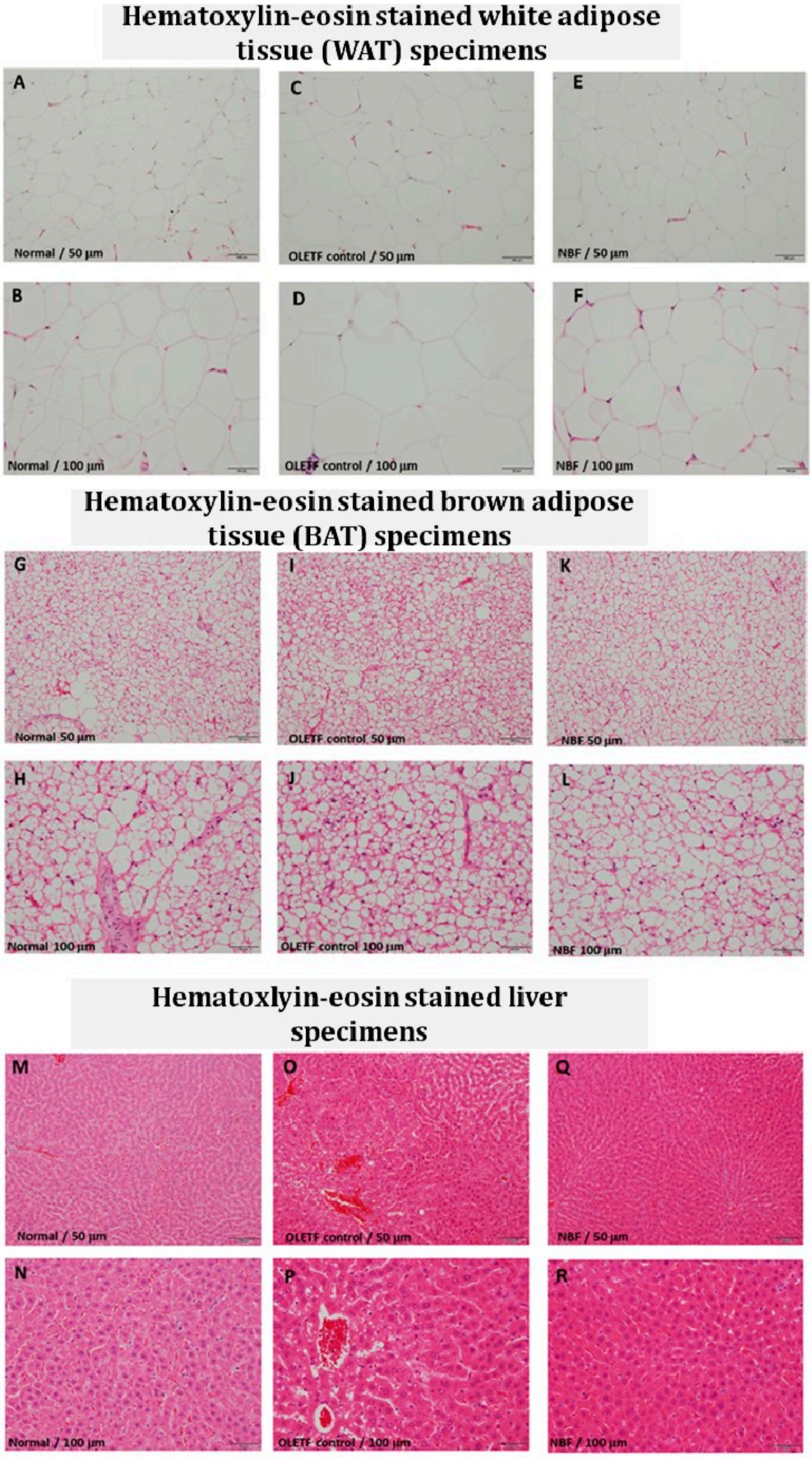

Supplementary Figure S1. Histopathologic changes in rat white/brown adipose tissue and liver specimens following NBF2 treatment. Legend: OLETF, Otsuka Long-Evans Tokushima Fatty rats; LETO, Long-Evans Tokushima Otsuka rats; BAT, brown adipose tissue; WAT, white adipose tissue; LIV, liver tissue
